# Mild malformation of cortical development with oligodendroglial hyperplasia in epilepsy (MOGHE): genetics, mechanisms and precision therapy

**DOI:** 10.1007/s00401-026-03059-6

**Published:** 2026-08-02

**Authors:** James Spyrou, Paul J. Lockhart, Snezana Maljevic, Katherine B. Howell, Christopher A. Reid

**Affiliations:** 1 The Florey Institute of Neuroscience and Mental Health, Kenneth Myer Building, 30 Royal Parade, Parkville, VIC 3052 Australia; 2 Faculty of Medicine, Dentistry and Health Sciences, The University of Melbourne, Parkville, VIC Australia; 3 Bruce Lefroy Centre for Genetic Health Research, Murdoch Children’s Research Institute, Parkville, VIC Australia; 4 Department of Paediatrics, The University of Melbourne, Parkville, VIC Australia; 5 Department of Neurology, Royal Children’s Hospital, Parkville, VIC Australia; 6 Epilepsy Research Centre, Department of Medicine, University of Melbourne, Austin Health, Heidelberg, VIC Australia

**Keywords:** SLC35A2, Somatic mosaicism, N-glycosylation, mTORopathies, Epileptogenesis

## Abstract

Mild malformation of cortical development with oligodendroglial hyperplasia in epilepsy (MOGHE) is a recently defined malformation of cortical development that is an important cause of childhood-onset drug-resistant epilepsy. Clinically, the epilepsies associated with MOGHE are heterogeneous, with infantile epileptic spasms syndrome (IESS) being the most common manifestation. Histopathologically, MOGHE demonstrates subtle cortical dyslamination, heterotopic neurons in the white matter, hypomyelination, and a distinctive increase in the density and clustering of oligodendroglial cells, features that distinguish it from other malformations of cortical development such as focal cortical dysplasia (FCD). Recent genetic analyses of epileptogenic tissue resected from individuals with MOGHE have identified somatic mosaic loss-of-function variants in *SLC35A2*. This gene encodes the Golgi transmembrane UDP-galactose transporter, suggesting disrupted N-glycosylation as a distinct pathogenic mechanism underlying epilepsy in this disorder. In this review, we present the current clinical, histopathological, and molecular understanding of MOGHE, with a particular focus on recent insights gained from experimental rodent and human cellular models of SLC35A2 deficiency. We contextualise these findings against established models of mTORopathies including FCD type 2, placing MOGHE within the broader malformation of cortical development spectrum. Synthesising this evidence, we observe that neuronal activity in models of both MOGHE and mTORopathies such as FCD type 2 converge on reduced action potential firing, despite their distinct genetic aetiologies. Finally, we discuss how these findings inform our understanding of epileptogenesis, especially the emergence of infantile epileptic spasms, and the development of future precision therapeutic strategies across malformations of cortical development.

## Introduction

Malformations of cortical development (MCD) are a heterogeneous collection of rare neurodevelopmental disorders that are a significant cause of paediatric refractory epilepsy and developmental delay [7, 30, 43, 63, 87, 89]. MCD are characterised by structural abnormalities resulting from perturbations in cortical formation and are present in approximately 40% of children with drug-resistant epilepsy [59, 64, 90]. MCD can be underpinned by genetic and environmental causes; however, it has been increasingly recognised that genetic etiologies account for the majority of cases [7, 14, 26, 41–43, 70, 85, 89, 93, 96]. The onset, severity, and clinical manifestations of epilepsy in cases of MCD are heterogeneous, in part depending on the type and severity of the malformation [89, 100]. MCD are often resistant to anti-seizure medications (ASMs), with surgical resection of affected brain tissue being the primary treatment option for many patients [44, 84, 108].

MCD can affect the cortex diffusely, or focally, in a uni- or multi-focal manner. Examples of focal MCD include focal cortical dysplasia (FCD) type 2, tuberous sclerosis complex (TSC), and mild malformation of cortical development with oligodendroglial hyperplasia in epilepsy (MOGHE). FCD and TSC are established clinico-pathological entities with a well-defined genetic and molecular underpinning; pathogenic variants in genes involved in the PI3K–AKT–mTOR signalling pathway (including *MTOR, RHEB, DEPDC5, STRADA, PIK3R2, PIK3CA, AKT, NPRL2, NPRL3, PTEN, TSC1,* and *TSC2*) have been shown to cause FCD type 2 and TSC, collectively termed ‘mTORopathies’ [10, 19, 38, 40, 54, 62, 66, 81, 83, 92]. MOGHE, however, has only recently been recognised as a distinct malformation, and mechanistic understanding remains limited.

MOGHE was first described in 2017 and, like other forms of MCD, is strongly associated with childhood-onset drug-resistant epilepsy [98]. MOGHE is characterised by histopathological hallmarks including heterotopic neurons in the white matter, clustering and increased density of oligodendroglial cells in both the subcortical white matter and deep layers of the cortex, and areas of reduced myelination and blurring of the grey-white matter boundary [12, 98]. In 2021, somatic pathogenic variants in *SLC35A2* were identified in brain tissue resected from individuals with MOGHE [12]. The *SLC35A2* gene is located at chromosome Xp11.23 and encodes the uridine diphosphate (UDP)-galactose transporter, involved in N-glycosylation and galactose transport [12]. Mosaic loss-of-function variants are thought to underpin the pathogenesis of MOGHE and its associated phenotype; however, the precise mechanisms by which an abnormality of glycosylation results in MCD and epilepsy remain unknown.

MOGHE and FCD type 2 have overlapping clinical presentations, especially that of infantile epileptic spasms syndrome (IESS) being a common epilepsy manifestation [5, 12, 16, 38, 72]. However, despite this convergence, they differ in their genetic basis, histopathological hallmarks, and molecular and electrophysiological drivers of seizure activity. A comparative analysis of MOGHE and FCD type 2 is, therefore, essential to clarify their nosological relationship within the wider context of FCD and MCD.

This review examines the molecular and functional consequences of pathogenic variants in *SLC35A2* and in genes of the mTOR signalling pathway. Particular emphasis is placed on recent findings from rodent and human cellular models of *SLC35A2* deficiency, compared with established models of TSC/FCD type 2. We first provide an overview of the evolving clinical landscape of MOGHE, and its similarities and differences with FCD. We then examine the role of SLC35A2 in glycosylation, and how perturbed galactose transport underpins the unique pathogenesis of MOGHE, contrasted with the role of mTOR-pathway dysregulation in TSC/FCD type 2. We then discuss how modelling studies using rodent and iPSC-derived neuron systems provide valuable mechanistic insight into MOGHE epileptogenesis and histopathology. We explore how these distinct genetic perturbations converge on common cellular phenotypes whilst producing the divergent histopathological manifestations of MOGHE and TSC/FCD type 2. Finally, we discuss pharmacological treatment approaches and how findings from models of both MOGHE and TSC/FCD type 2 inform the development of precision therapies for specific molecular genetic underpinnings, as well as treatments with potential utility across the MCD spectrum. This article presents a narrative review and is, therefore, intended to provide a thematic synthesis of the current literature rather than a systematic review of all published studies.

## Malformations of cortical development: a complex and evolving spectrum

In contrast to the more comprehensively studied FCD type 2 and TSC, other focal MCD are less well understood. Early classification frameworks recognised that some patients displayed subtle disruptions in the cytoarchitecture of the cortex, without the dysmorphic neurons (with or without balloon cells) that are characteristic of the mTORopathies. These more ‘subtle’ MCD include FCD type 1, in which disruption to cortical lamination is present, and mild malformation of cortical development (mMCD), in which lamination typically appears normal, but heterotopic neurons are identified in the subcortical white matter [11, 86]. Patients with these MCD are diagnostically challenging. For mMCD in particular, lesions are often undetectable or very subtle on neuroimaging, and this label was typically only assigned after histological analysis of resected brain tissue from epilepsy surgery, with identification of heterotopic neurons in the subcortical white matter [11, 86]. It was recognised that the mMCD diagnostic label likely encompassed a range of pathological entities, potentially underpinned by distinct biological aetiologies. However, without distinguishing molecular, histological, or genetic criteria, clearer delineation of this group was not possible. Therefore, mMCD functioned as an interim, umbrella category describing subtle but epileptogenic cortical pathology, awaiting further in-depth genetic and molecular characterisation.

MOGHE as a clinico-pathological entity emerged from the broad and heterogeneous category of mMCD when it was first described in 2017 [98]. At that time, the defining histopathological features of MOGHE were recognised within cases broadly classified under the mMCD spectrum. This highlighted a previously unappreciated subgroup of mMCD histologically defined by the presence of increased Olig2-positive oligodendroglial cells in the white matter and deep cortical layers [98]. A unifying genetic basis for these cases was discovered when recurrent somatic variants in *SLC35A2* were identified through subsequent genetic analyses across people diagnosed with mMCD and a subset of those diagnosed with FCD type 1 [12]. This convergence of histopathological and genetic evidence led to the reclassification of these cases under the entity of MOGHE, which is now recognised as a distinct entity in the current classificatory framework of MCD [78]. However, the subtle histopathological features and evolving molecular definition of MOGHE have historically contributed to diagnostic uncertainty and under-recognition, underscoring the need for integrated clinico-pathological and mechanistic frameworks.

## Clinical presentation of MOGHE

Epilepsy is typically the presenting clinical feature of MOGHE. Although seizure onset is usually in infancy or early childhood, onset as late as 24 years has been reported [16, 37, 69]. The epilepsies associated with MOGHE are heterogeneous, but frequently are an epileptic encephalopathy. Seizure types are variable, including epileptic spasms and focal seizures; in those with focal seizures, a frontal lobe seizure predilection has been noted [37, 42]. Electroencephalography (EEG) typically shows frequent interictal epileptiform discharges (IEDs), and can be ‘circumscribed’ (i.e. confined to the malformed region) or ‘widespread’ [37]. One study reported two ‘peculiar’ IED patterns, which were more prominent in older patients: intermittent, unilateral 2–2.5Hz slow spike-wave paroxysms in wake, and more frequent and polymorphic high-amplitude IEDs at 0.5–1.5Hz in sleep [37]. Overall, IESS is the most common epilepsy syndrome. Where seizure onset is after infancy, the epilepsy may be a drug-resistant focal epilepsy [12, 98].

The MRI features of MOGHE can be subtle, making a clinical (i.e. pre-operative) diagnosis of MOGHE challenging. Brain MRI may show blurring of the grey-white matter boundary or an increased T2 and fluid-attenuated inversion recovery (FLAIR) signal in the subcortical white matter; however, some cases are MRI-negative [12, 45, 116]. The size of the malformed area varies, although many people with MOGHE have lobar, multilobar, or even hemispheric malformations.

In most people with MOGHE, seizures are refractory to ASM treatment. However, the outcomes of resective or disconnective epilepsy surgery are generally favourable. Approximately two-thirds of cases are seizure-free postoperatively with a median follow-up of 2 years, with many with longer durations of follow-up remaining seizure-free [116].

To date, *SLC35A2* is the only gene associated with MOGHE. Genetic testing on resected brain tissue identifies a mosaic *SLC35A2* variant in over 70% of people diagnosed with MOGHE [116]. Currently, there is no evidence of a correlation between the specific type of *SLC35A2* mosaic variant (nonsense, missense, frameshift) or the variant allele frequency with malformation size, epilepsy severity, or surgical outcomes [116].

In people with MOGHE, development prior to the onset of epilepsy is typically normal or near normal. Ultimately, however, developmental and cognitive impairments are seen in most people, and comorbidities such as autism spectrum disorders are common. Developmental and cognitive impairments can be severe, particularly in those with a younger age of seizure onset, or longer seizure duration. Impairments often persist despite successful resective surgery controlling seizures. However, younger age at surgery is strongly associated with more favourable developmental outcomes, highlighting that early successful seizure treatment can lead to improved post-operative outcomes for patients with MOGHE, by limiting the progressive developmental impacts of uncontrolled epilepsy [6, 27, 69].

## Genetic and molecular underpinnings of MOGHE

Building on the development of MOGHE as a distinct clinico-pathological entity, subsequent studies in additional cohorts confirmed *SLC35A2* mosaicism as the primary cause of MOGHE [6, 56, 69]. Variant allele frequencies (VAF%) of somatic variants in *SLC35A2*, measured using droplet digital polymerase chain reaction (ddPCR) or next-generation sequencing of genomic DNA from resected tissue, range from as low as 1.0% to as high as 52.6%. Notably, *SLC35A2* pathogenic variants are enriched in clustered oligodendroglial cells that express Oligodendrocyte transcription factor 2 (*Olig2*) [12], a basic helix–loop–helix transcription factor and oligodendroglial lineage marker [77, 119]. *SLC35A2* variants are also enriched in heterotopic neurons relative to grey matter cortical neurons [12]. No correlation has been identified between VAF% or variant type in *SLC35A2* and the severity of symptoms or extent of malformation [6, 12,13, 16].

### Glycosylation and the canonical role of SLC35A2

**Fig. 1:**
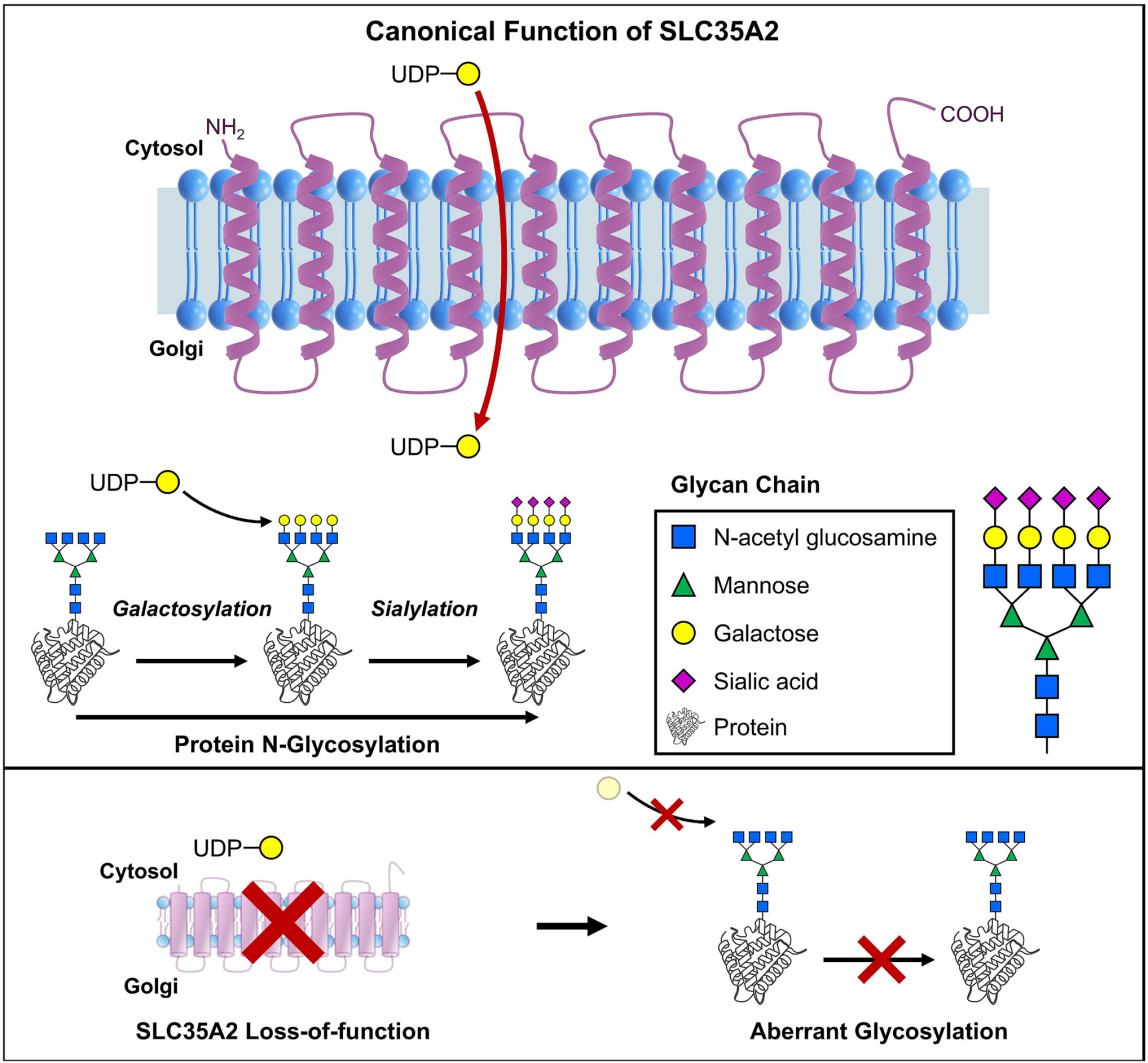
Canonical function of SLC35A2. The SLC35A2 protein is responsible for the transport of uridine diphosphate (UDP)-galactose from the cytosol into the Golgi apparatus, where this transported galactose is then attached onto the growing glycan chains of glycosylated proteins by galactosylation. These galactose residues on glycan chains can then be sialylated by the addition of terminal sialic acid residues. Loss of SLC35A2 function results in the deficiency of available galactose within the Golgi apparatus, causing glycosylation to be perturbed in affected cells due to the lack of galactose residues on glycosylated proteins

*SLC35A2* encodes the uridine diphosphate (UDP)-dependant galactose transporter (UGT), which mediates the transport of galactose from the cytosol into the lumen of the Golgi apparatus, a critical step in the glycosylation of proteins and lipids (Fig. 1). Within the Golgi, galactosyltransferase incorporates the galactose residues into elongating glycan chains [18, 33]. There are two primary forms of glycan chains: N-linked glycans, which are attached to the nitrogen atom of an asparagine side chain, and O-linked glycans, which are attached to the oxygen atom of a threonine, serine, hydroxylysine, or hydroxyproline side chain [109]. Glycosylation of proteins is important for a wide array of critical biological processes, including protein structure, folding and stability, and protein localisation [99]. Galactose residues are key component of N-linked glycan chains. In the brain, N-linked glycans modulate critical processes including maintenance of resting membrane potential, axon firing, and neurotransmitter release; disorders of glycosylation disrupt these processes and impair neuronal function [17, 35]. Abnormal glycosylation also leads to aberrant sialylation, the addition of sialic acid units to the terminal end of glycan chains, most often to galactose residues. Sialylation regulates cell fate decision-making, including during embryogenesis and neurodevelopment [65]. Sialylated sphingolipids (gangliosides) modulate neuronal excitability and myelin–axon interactions [97]. Furthermore, polysialic acid, long (> 90 unit) polymers of sialic acid attached to glycoproteins, is present in the radial glia of the developing cortex and has an important role in neuroblast migration [97]. Collectively, glycosylation and sialylation are indispensable for normal neurodevelopment and brain cell function. In cells with a loss-of-function variant in *SLC35A2*, galactose is not efficiently transported into the Golgi, and galactose residues are not properly incorporated into glycan chains, resulting in truncated glycans and dysregulated sialylation.

Neural cell adhesion molecules (NCAMs) are glycoproteins that are key regulators of neurodevelopment [2, 24]. Highly polysialylated in the developing brain, NCAMs regulate neuroprogenitor migration, and neurito- and dendrito-genesis [24]. Aberrant sialylation of NCAMs due to SLC35A2 loss-of-function, together with broader disruption of glycosylation across neurodevelopmental pathways, may converge on impaired neuronal migration and cortical lamination, providing a mechanistic link with the disorganised cortical architecture observed in MOGHE. Aberrant sialylation of NCAMs due to *SLC35A2* loss-of-function may therefore contribute to the histopathology of MOGHE.

In summary, UGT plays a central role in maintaining galactose availability for N‑glycosylation within the Golgi apparatus. Loss of this function is likely to impact neuronal and glial development by disrupting glycoprotein function and trafficking, impairing sialylation-dependent processes such as cell migration and differentiation, and altering neuronal excitability. This molecular framework links mosaic *SLC35A2* dysfunction to both the histopathological and epileptogenic features of MOGHE, but does not tell us which specific mechanisms play a critical role in MOGHE’s pathogenesis.

## Insights into MOGHE from experimental models

**Fig. 2:**
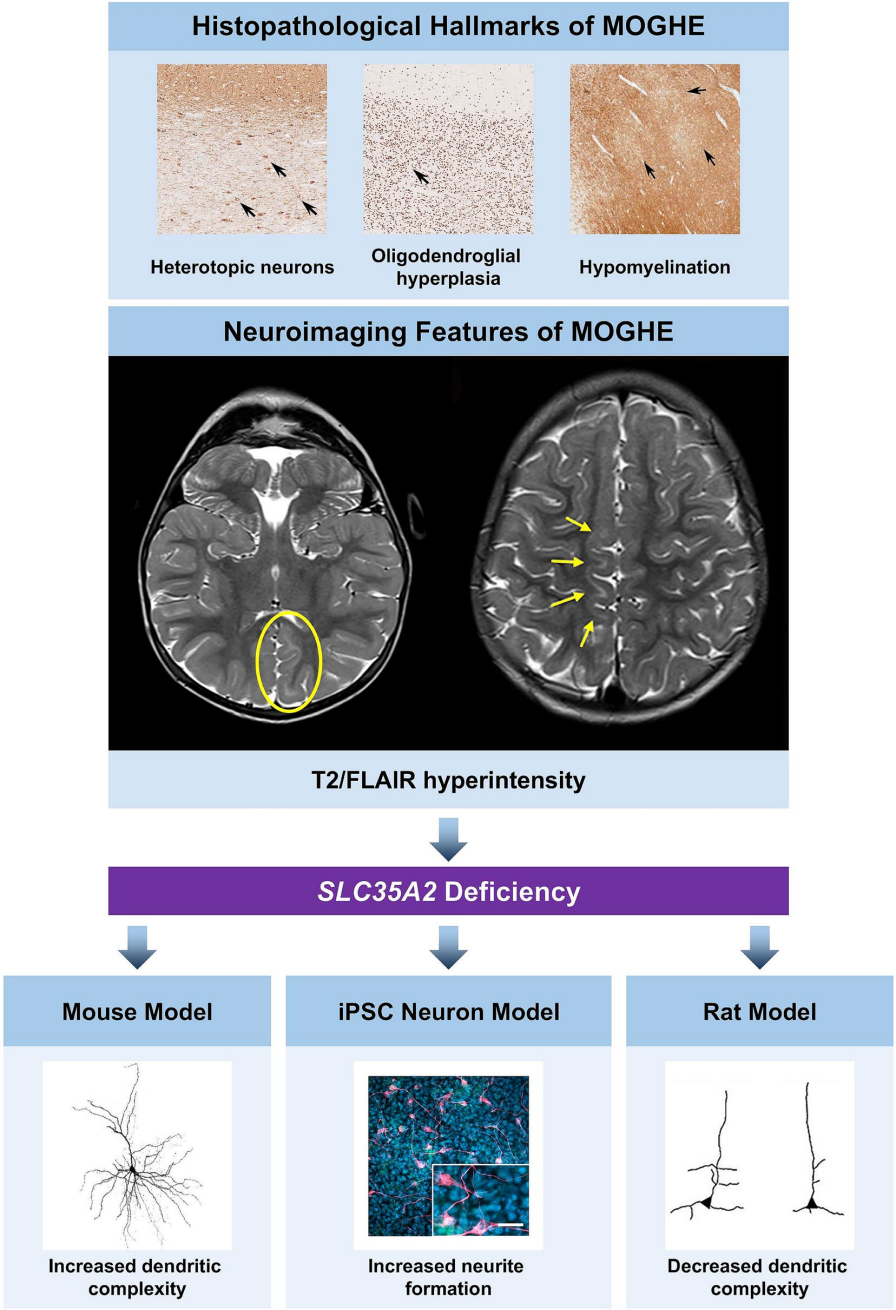
MOGHE is characterised by heterotopic neurons in the white matter (black arrows), dense clusters of Olig^2+^ oligodendroglial cells (black arrow), and hypomyelination (black arrows) in surgically resected brain tissue. Neuroimaging demonstrates characteristic T2/FLAIR hyperintensity at the corticomedullary junction (yellow circle/arrows). Preclinical models of *SLC35A2* deficiency indicate altered neuronal development, with mouse and human iPSC-derived neurons showing increased dendritic complexity and neurite formation, respectively, whereas rat model neurons show reduced dendritic complexity. Images were adapted from [12, 16, 31, 60, 71, 103] (colour figure online)

Mouse models of TSC and FCD2B that recapitulate somatic mosaicism of mTOR-pathway variants have been instrumental in elucidating the mechanisms of epileptogenesis in these FCMs [50,51, 81]. More recently, mouse, rat, and human iPSC-derived neuronal models have been developed to study MOGHE and *SLC35A2* deficiency. These models are beginning to provide insights into how *SLC35A2* loss-of-function contributes to cortical malformations, oligodendroglial hyperplasia, and altered network excitability (Table 1; Fig. 2).

**Table 1:** List of experimental models of *SLC35A2* deficiency

Species/model	Method/variant	Cell type	Key histological phenotype	Seizure phenotype/electrophysiology	References
Mouse	CRISPR/shRNA mosaic KO (IUE)	Excitatory cortical neurons	Heterotopic neurons, increased dendritic arborisation	Increased seizure susceptibility, increased epileptiform spiking on EEG, reduced neuronal excitability	[29, 103]
Mouse	Conditional KO (Emx1-Cre)	Forebrain neurons + oligodendroglia + astrocytes	Delayed radial migration, dendritic abnormalities, oligodendroglial hyperplasia	Spontaneous seizures, early mortality	[114]
Mouse	Conditional KO (Olig2-Cre)	Oligodendroglia	Oligodendroglial hyperplasia only	Spike bursts on EEG, no spontaneous seizures	[114]
Mouse	Conditional KO (Olig2-Cre)	Oligodendroglia	Oligodendroglial hypoplasia in older animals, severe hypomyelination, reactive gliosis	Spontaneous seizures, frequent interictal spiking	[8]
Rat	Patient variant expression/shRNA-mediated silencing (IUE)	Excitatory cortical neurons	Heterotopic neurons, reduced dendritic arborisation	*Not investigated*	[31]
Human iPSC	CRISPR patient variants / KO	Neurons	Impaired differentiation and aberrant neurite morphology	Reduced firing, increased bursting, asynchronous network activity	[60]

*iPSC* induced pluripotent stem cells, *shRNA* short hairpin RNA, *KO* knockout, *IUE *in utero electroporation, *EEG* electroencephalography

### Mouse models of Slc35a2 deficiency

In utero electroporation-mediated mosaic knockout or knockdown in excitatory neuronal progenitors, performed by us and Elziny et al*.*, has produced mouse models that recapitulate key histopathological hallmarks of MOGHE [29, 103]. These models have enabled investigation of how impaired galactose transport in neurons contributes to MOGHE pathogenesis.

Elziny et al*.* employed both CRISPR/Cas9 mosaic knockout and short hairpin RNA (shRNA)-mediated knockdown of *Slc35a2* to model MOGHE. Both methodologies resulted in impaired radial neuron migration and disrupted corticogenesis, recapitulating the hallmark heterotopic neurons observed in MOGHE patient tissue [29]. *Slc35a2* mosaic KO mice did not display spontaneous seizure activity. However, following subcutaneous administration of the proconvulsant pentylenetetrazole (PTZ), which reliably evokes convulsive seizures, they showed a decreased seizure threshold and greater seizure severity compared to control mice. Similarly, our CRISPR/Cas9-mediated mosaic *Slc35a2*-knockout mouse model displayed heterotopic neurons in the white matter, and increased epileptiform activity on electrocorticography (ECoG) in the absence of spontaneous seizures, in addition to behavioural hyperactivity [103].

Extending these findings, Yoon et al*.* generated two conditional knockout mouse lines using a novel ‘floxed’ *Slc35a2* allele [114]. Transgenic mice with floxed alleles are engineered, such that the gene of interest is flanked by loxP sites, permitting its excision only where Cre recombinase is expressed, resulting in a conditional knockout of a specific gene in defined cell lineages. In the Emx1-Cre line, *Slc35a2* was deleted in excitatory neuronal and oligodendroglial progenitors of dorsal telencephalic lineage, resulting in broad forebrain knockout of *Slc35a2*. These mice demonstrated abnormalities of cortical development, delayed radial migration of neurons from the subventricular zone, impaired dendritic maturation, and abnormal neuronal excitability [114]. Notably, there was increased oligodendroglial density, recapitulating a defining feature of MOGHE, consistent with patient histopathology. In addition, Emx1-Cre cross mouse line showed both early onset spontaneous seizures and early mortality. In contrast, the Olig2-Cre cross mouse line, in which *Slc35a2* deletion was restricted to oligodendroglia, demonstrated increased oligodendroglial density and exhibited spontaneous bursting of ictal spikes on EEG recordings, but did not develop spontaneous seizures or display aberrant neuronal migration [114]. However, a recent Olig2-specific *Slc35a2* conditional knockout mouse model generated by Bartel et al*.* showed a more severe phenotype, with oligodendrocyte-specific *Slc35a2* loss-of-function resulting in severe hypomyelination, reactive gliosis, and reduced oligodendrocyte numbers in the corpus callosum, accompanied by spontaneous seizures and frequent interictal spiking [8]. Together, these conditional knockout models suggest that neuronal *Slc35a2* loss contributes to the malformation of cortical development and to seizure initiation. On the other hand, Oligodendroglial *Slc35a2* deficiency independently contributes to seizure generation independent of an impact on cortical development.

Oligodendroglial hyperplasia, a defining histopathological feature of MOGHE, may reflect perturbed lineage development and myelin homeostasis rather than simply elevated proliferation. This is reinforced by oligodendroglial conditional knockout mouse lines showing increased oligodendroglial cell density in the subcortical white matter along with a reduction of oligodendrocytes in the corpus callosum, suggesting altered migration being a potential factor [8]. Given the critical role of these cells in myelin formation and maintenance, impaired oligodendroglial maturation provides a plausible mechanistic explanation for the hallmark hypomyelination consistently observed in the white matter of resected MOGHE tissue [52, 57]. Notably, hypomyelination downstream of oligodendrocyte dysfunction has also been observed in mTORopathies [39], with hypo- or demyelination potentially promoting epileptogenesis [25]. Collectively, these data suggest a complex interplay between dysfunction in neurons and oligodendroglial cells as a driver of excitability in MOGHE.

### Rat models of MOGHE

Complementing findings from mouse studies, Falace et al*.* established rat models of MOGHE by in utero electroporation of patient-derived pathogenic *SLC35A2* variants and by shRNA-mediated silencing of rat *Slc35a2* [31]. These pathogenic variants were the missense variant c.844G > A (p.Gly282Arg) and the frameshift variant c.837_847del (p.Phe280Thrfs^*^10), both previously identified in individuals with MOGHE [6]. Rat cortical neuronal progenitors were transfected on embryonic day E15.5, a developmental stage selected as Slc35a2 levels increase between E15 and postnatal day P1, and remain stable thereafter [31]. Overexpression of these pathogenic variants disrupted neuronal migration to a similar extent as shRNA-mediated knockdown. Approximately one-third of migrating neurons failed to reach the cortical plate at the late embryonic stage (E20), whereas almost all migrating neurons in control animals were correctly localised. At later postnatal stages (P6 and P15), a significant proportion of GFP-positive transfected neurons were heterotopic, incorrectly located in the deep layers of the cortex (L5/6) or in the white matter itself. These findings illustrate that impaired neuronal migration and the resulting white matter heterotopias, key pathological features of MOGHE, are conserved across rat, mouse, and human systems. Most importantly, these models demonstrate that *Slc35a2* deficiency in a subset of pyramidal cortical neurons alone is sufficient to disrupt neuronal migration and cortical cytoarchitecture, consistent with human pathology. In addition, *Slc35a2* deficiency contributes to seizure susceptibility and a hyperexcitable phenotype [29, 103].

However, there are some key differences observed between these models. Most notably, in the rat models, successfully transfected pyramidal neurons that migrated to the correct cortical layer displayed reduced dendrite outgrowth and processes compared to neurons transfected with control scramble-shRNA [31]. In contrast, in our CRISPR-mediated mosaic knockout mouse model, individual transfected *Slc35a2*-KO pyramidal neurons correctly located in layer 2/3 of the cortex (i.e. ‘normotopic’) displayed an increase in dendritic branching and complexity [103]. Several factors may account for these divergent morphological findings. Rat neurons were imaged at P6 via endogenous GFP expression and NeuN staining, whereas mouse neurons were filled with biocytin at P20-33 and stained with a streptavidin–fluorophore conjugate for imaging [31, 103]. Differences in species, developmental timepoints, and imaging modality may therefore contribute to the distinct dendritic phenotypes observed between the mouse and rat models.

### Stem cell-derived models

In addition to rodent models, human cell culture-based models have demonstrated that *SLC35A2* deficiency alters neuronal differentiation and network behaviour. Lai et al*.* reprogrammed fibroblasts from a healthy male and introduced loss-of-function variants in *SLC35A2* via CRISPR/Cas9-editing [60]. Isogenic human-induced pluripotent stem cell (iPSC) lines were generated with the loss-of-function (LOF) missense variant c.910 T > C (p.Ser304Pro; *SLC35A2*^S304P/Y^) or complete *SLC35A2* knockout (*SLC35A2*^−/Y^) [110], and differentiated into neurons using a modified dual SMAD inhibition protocol. A key advantage of this model was the ability to accurately characterise glycomic signatures by isolation, purification and high-performance liquid chromatography (HPLC) analysis of N-glycans. Equivalent analyses are not feasible in a mosaic animal model [60]. The electrophysiological characteristics of the differentiated neurons were also measured by whole-cell patch clamp and multi-electrode array (MEA) recording. HPLC revealed profound alterations in glycosylation in both the *SLC35A2* loss-of-function and knockout lines. There were significant reductions in the complexity of N-glycan chains, with bi-, tri- and tetra-antennary (i.e. containing 2, 3 or 4 branches) galactosylated N-glycans being largely absent. These glycosylation defects were observed in undifferentiated iPSC lines and after 30 days of neuronal differentiation. The N-glycan profiles of *SLC35A2*^S304P/Y^ and *SLC35A2*^−/Y^ lines were virtually identical, supporting the conclusion that the pathogenic mechanism associated with missense variants in MOGHE is loss-of-function. These findings align closely with N-glycosylation defects, including glycoform and glycoprotein abnormalities, identified using glycan labelling and intact glycopeptide profiling of resected lesional tissue from individuals with MOGHE [68]. Aberrant brain signatures were significantly enriched for cell adhesion gene ontology (GO) and Kyoto Encyclopedia of Genes and Genomes (KEGG) pathways.

Neurogenesis in *SLC35A2*^S304P/Y^ and *SLC35A2*^−/Y^ cell lines was also dysregulated, with affected neurons showing early neurogenesis and increased neurite length at day 12 of differentiation. In addition, MEA recordings showed significantly reduced firing rates, with increased bursting and asynchronous firing [60]. Furthermore, differentiation of *SLC35A2* loss-of-function lines was shown to be skewed towards a more GABAergic fate. Differences in neural composition and excitatory/inhibitory imbalance were supported by immunocytochemical and gene expression analysis, and by MEA recordings. Pharmacological experiments further highlighted network differences associated with *SLC35A2* loss-of-function. Administration of the AMPA/kainate receptor antagonist cyanquixaline (CNQX) caused control networks to become asynchronous, recapitulating the asynchronous baseline firing of *SLC35A2* loss-of-function networks, which, however, were unaffected by CNQX. In contrast, treatment with the GABA_A_ antagonist bicuculline increased the synchrony of *SLC35A2* loss-of-function networks, although the rescue was not to the level of control networks [60]. Although these iPSC-derived neuron lines lack the architectural complexity of the developing cortex, these models provide important mechanistic insight into how *SLC35A2* mosaicism may contribute to cortical malformation and epileptogenesis.

## MOGHE and focal cortical dysplasia type 2

FCD is a spectrum of highly variable, localised malformations of cortical development that are strongly associated with refractory epilepsy and cytoarchitectural perturbations of the cortex [11, 20, 28, 32, 34, 106]. It is important to delineate the similarities and differences between MOGHE and other FCM, particularly well-characterised entities such as FCD type 2, to clarify points of overlap and divergence in their cellular and molecular pathology.

FCD type 2 displays severe cortical dyslamination and a blurred grey-white matter junction. In addition, cytomegalic dysmorphic neurons are present in the cortex. FCD type 2 is classified into two subtypes: Type 2B where balloon cells are, and Type 2A in which they are absent [11]. Somatic variants occurring in the brain during neurodevelopment are a major cause of FCD Type 2. These variants can perturb regulation of the PI3K/AKT/mTOR signalling cascade, resulting in significantly increased activity of mechanistic target of rapamycin complex 1 (mTORC1). This protein complex regulates several key cellular processes including cell growth, proliferation, differentiation, and autophagy [22, 112, 118]. Increased mTOR signalling, termed *mTOR hyperactivation* [21, 75], can result from the dysregulation of multiple proteins in the pathway [81]. mTOR kinase, encoded by *MTOR*, is the catalytic component of mTORC1 and mTORC2 [61], and somatic missense variants in mTOR are a well-established cause of FCD type 2 [66,67, 79]. In addition, *TSC1* and *TSC2*, which encode hamartin and tuberin, respectively, form the TSC complex. This complex negatively regulates mTORC1 activity by inhibiting the GTPase *Ras homolog enriched in brain* (Rheb), a key driver of mTORC1 activity [73, 107]. Pathogenic germline variants in *TSC1* and *TSC2* cause tuberous sclerosis complex (TSC), a multisystem disorder characterised by brain malformations, severe lifelong epilepsy and developmental delay, as well as tumours of the brain, skin, lungs, and kidneys. These features are due to disruption of TSC complex function, resulting in constitutive activation of mTORC1 [23, 46]. As with other MCD, surgery represents primary treatment for the majority of affected individuals [9, 32, 47, 55, 102]. Surgical outcomes are generally favourable, with many individuals achieving seizure freedom following resection [88, 94, 113, 117].

Cytomegalic dysmorphic neurons are a defining histopathological feature of both TSC and FCD Type 2 [4, 23, 48]. These neurons ectopically express the ion channel HCN4 (hyperpolarisation-activated cyclic nucleotide-gated potassium channel isoform 4) in both resected patient tissue and mouse models of mosaic mTOR hyperactivation. Electroporation-based mouse models have suggested that this ectopic expression is likely to be a primary driver of seizures and epileptic activity in these conditions [15, 51, 80,81, 104]. In TSC/FCD2B model mice, rapamycin, the mTOR-pathway inhibitor, administered every 48 h from postnatal day 1–2 months of age, prevents the development of dysmorphic neurons, ectopic HCN4 channel expression, and spontaneous seizures. These findings indicate that HCN4 channel expression precedes seizure onset and is dependent on mTOR-pathway hyperactivation [51]. In addition, blocking HCN4 channel activity in dysmorphic neurons, by co-transfecting electroporated mice with a non-functional HCN4 channel construct in addition to an mTOR-hyperactivating construct, prevents the development of epilepsy in this model [51].

**Fig. 3:**
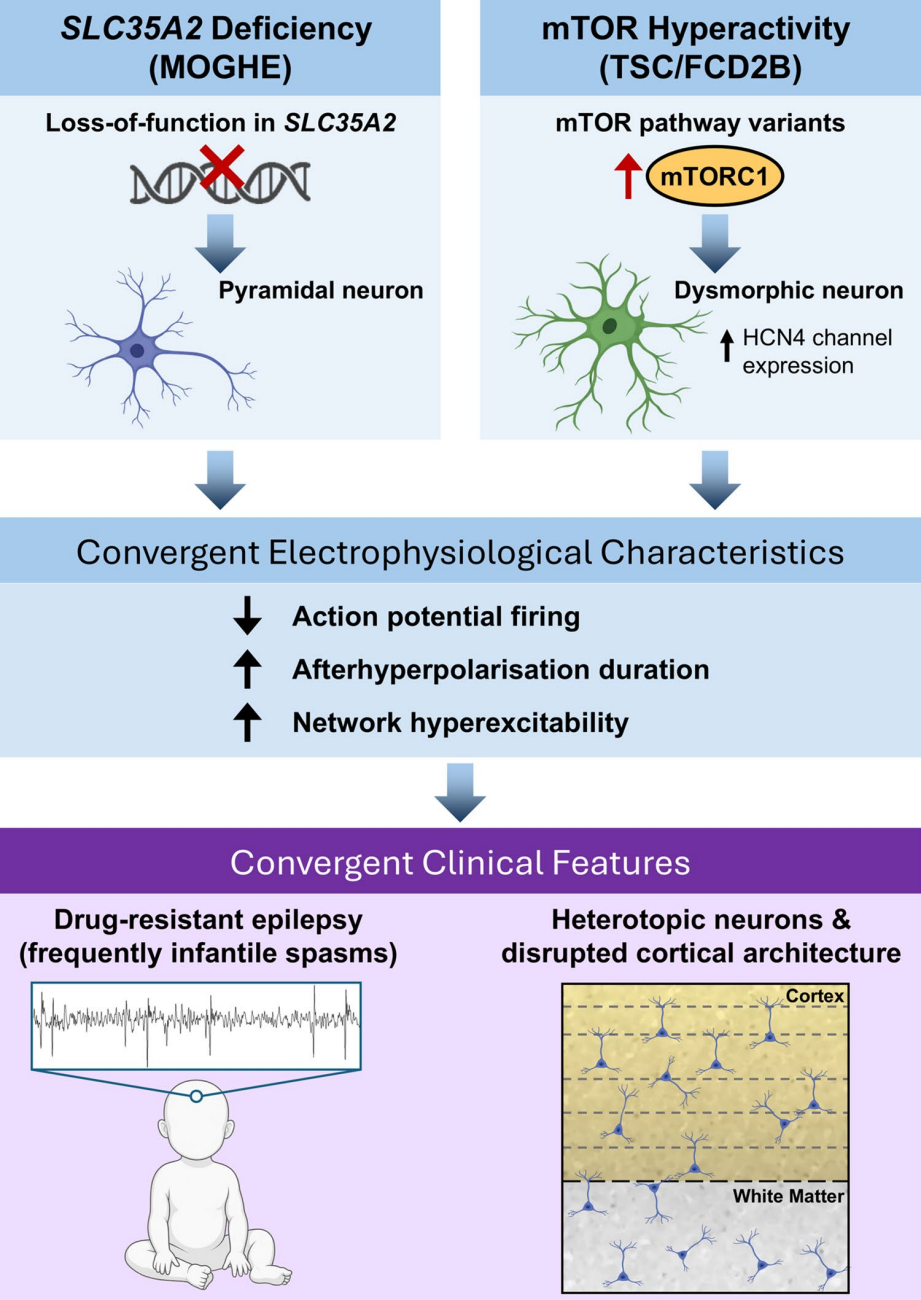
An illustration of key electrophysiological characteristics and clinical features that show convergence between *SLC35A2* deficiency (MOGHE) and mTOR hyperactivation (TSC/FCD type 2)

Clinically, MOGHE and FCD type 2 share several features, including early onset, drug-resistant epilepsy, and IESS being a common presentation of epilepsy [16, 71]. Histopathologically, both disorders exhibit cortical dyslamination and heterotopic neurons. However, MOGHE is distinguished by prominent increased density and clustering of oligodendroglial cells and the absence of cytomegalic dysmorphic neurons that define FCD type 2 [12, 98]. Importantly, despite their different genetic and molecular underpinnings, electroporation-based mouse models of *Slc35a2* deficiency and mTOR hyperactivity reveal functional convergence, as discussed below. Comparisons of clinical, histological, and model-based convergences and divergences of MOGHE and FCD type 2 are summarised in Table 2, with convergences illustrated in Fig. 3.

**Table 2:** List of features of MOGHE and FCD type 2 in both patients and mouse models

Feature	MOGHE	FCD type 2	References
Molecular genetic cause	Mosaic *SLC35A2* loss-of-function	Mosaic mTOR-pathway hyperactivity	[12, 51]
Infantile epileptic spasms	**Common seizure manifestation**	**Common seizure manifestation**	[16]
Heterotopic neurons	**Present**	**Present**	[5, 12, 98]
Oligodendroglial hyperplasia	Prominent	Absent	[12, 98]
Hypomyelination	**Present**	**Can be present**	[12, 98]
Dysmorphic neurons	Absent	Present	[15, 51, 81]
HCN4 channel expression	Normal expression	Ectopic expression in dysmorphic neurons	[15, 51, 81]
Seizure activity in mouse models	Epileptiform spiking (IUE and Olig2-Cre mice), **spontaneous seizures** (Emx1-Cre mice)	**Spontaneous seizures**	[51, 103, 114]
Reduced action potential firing	**In affected neurons**	**In affected neurons**	[51, 103]

Bold text indicates convergent phenotypes shared between MOGHE and FCD type 2

## Functional convergence of *Slc35a2* deficiency and mTOR hyperactivity

Beyond the morphological changes observed in rodent models of *Slc35a2* mosaicism, individual normotopic *Slc35a2*-KO neurons in mice exhibit a distinctive electrophysiological phenotype. This phenotype includes reduced action potential firing at given injected currents, prolonged afterhyperpolarisation duration, and reduced burst-firing in comparison to control neurons [103]. A reduction in action potential firing differs from that which is observed in most other models of drug-resistant epilepsy, which show increased action potential firing. This distinction is notable, as it implicates alternative, potentially circuit-level mechanisms as underlying epileptogenesis rather than increased intrinsic excitability of affected neurons. This cellular phenotype is very similar to that observed in TSC/FCD2B model mice [51, 81], despite the fundamentally different genetic causes underlying these malformations. The cellular mechanisms that result in reduced action potential firing are not known. In mTORopathies, both human tissue and mouse models are characterised by dysmorphic neurons that ectopically express HCN4 channels, which are believed to be a key driver of seizures [51, 81]. However, this is unlikely to underlie this observed electrophysiological convergence, as dysmorphic neurons and ectopic HCN4 channel expression are absent in MOGHE and *Slc35a2*-deficient models. This independence is further supported by the observation that mouse neurons that have been co-transfected with an mTOR-hyperactivating construct and a non-functional Hcn4 channel construct also display reduced action potential firing [51].

Reduced firing rates accompanied by increased bursting have also been observed in *SLC35A2*^S304P/Y^ (missense loss-of-function) and *SLC35A2*^−/Y^ (hemizygous knockout) iPSC-derived neurons, which closely mirror the electrophysiological profile of *SLC35A2* knockout neurons from electroporated mouse models [60, 103]. A comparable electrophysiological phenotype has been observed in a mechanistically distinct context of channelopathies caused by *SCN2A* loss-of-function. *SCN2A* encodes the voltage-gated sodium channel Na_V_1.2 [74, 82, 105] and its haploinsufficiency is associated with infantile-onset seizures and refractory epilepsy [95]. *SCN2A*^±^ mice exhibit frequent ictal spikes on EEG following administration of the chemoconvulsant 4-aminopyridine (4-AP), a non-selective inhibitor of delayed rectifier potassium channels responsible for action potential repolarisation. This response is similar to the PTZ-induced spiking observed in *Slc35a2*-deficient mice [105]. Here, the reduction in neuronal excitability likely arises from the loss of sodium channels, a mechanism distinct from either mTOR hyperactivation or impaired N-glycosylation. As experimental models of these disorders reveal a convergent reduction in intrinsic action potential firing frequency, this suggests that epileptogenesis may emerge from disrupted cortical circuit development rather than from increased intrinsic excitability of individual pyramidal neurons.

The relevance of reduced (as compared to increased) firing, in regard to whether it may underpin different clinical features or have different treatment implications, is still unclear. One key common feature to these three disorders, MOGHE, TSC/FCD2B, and *SCN2A* loss-of-function-related epilepsy, despite their markedly different genetic aetiologies, is that infantile spasms are a common manifestation of seizures [49, 63, 116]. It would be of interest to investigate further into whether the observed reduction in action potential firing could represent an ‘electrophysiological signature’ that might be common across different causes of infantile spasms.

## Potential therapeutic avenues

The recent identification of aberrant glycan formation as a unique biomarker and likely mechanistic driver of MOGHE raises the possibility of targeted interventions aimed at restoring normal glycosylation or correcting downstream network abnormalities [1]. A recent series reported ᴅ-galactose supplementation in a series of 12 individuals with histopathologically confirmed MOGHE, 6 with pathogenic *SLC35A2* variants. Six individuals (2 with SLC35A2 variants) had ongoing seizures despite epilepsy surgery, and the remaining six did not have seizures but had cognitive impairments and ongoing epileptiform activity on EEG. In 3/6 individuals with seizures, galactose administration reportedly led to a ≥ 50% reduction in seizure frequency, which was maintained for 6 months of supplementation [1]. Reduction in epileptiform activity was demonstrated on 24-h video EEG for 2 of these 3 patients. Additionally, improvements in behaviour and/or cognition were reported in some patients; overall, 9/12 patients were deemed ‘global responders’ due to improvements in seizures and/or cognition. This study was small, unblinded and non-placebo-controlled, which prevents definitive conclusions being drawn. However, the findings provide a rationale for further investigation of galactose as a targeted intervention in *SLC35A2*-associated MOGHE, in a larger, blinded, placebo-controlled clinical trial. Demonstration that ᴅ-galactose supplementation can rescue the phenotypes in *Slc35a2* mosaic rodent models or correct the aberrant glycomic profiles of *SLC35A2* loss-of-function iPSC-derived stem cell models, would also provide valuable data to support further, larger human trials.

Epilepsy in MOGHE, and in TSC/FCD type 2, frequently presents as infantile epileptic spasms syndrome, a developmental and epileptic encephalopathy where seizures exacerbate perturbations in neurological development. Therefore, understanding and effectively treating infantile epileptic spasms across both TSC/FCD type 2 and MOGHE are critically important for minimising the ongoing impact of these seizures and optimising cognition and development. This applies to potential precision therapies, and also to use of existing anti-seizure medications. Individuals with *SCN2A* loss-of-function variants show poor responses or worsening of seizures in response to sodium channel blockers [111, 115]. Therefore, due to the similar reduction in neuronal firing observed in experimental models of MOGHE, TSC/FCD type 2, and *SCN2A* loss-of-function, one prediction is that sodium channel blocking anti-seizure medications may not be effective against infantile epileptic spasms in these syndromes [51, 74, 103, 105]. There is some clinical evidence that sodium channel blockers, including carbamazepine, oxcarbazepine, and phenytoin, may aggravate infantile epileptic spasms in TSC and other causes [53, 101]. It is important to note that this hypothesis pertains specifically to infantile epileptic spasms, as sodium channel blockers may be effective for treatment of focal seizures [72, 76]. Additional pre-clinical and clinical studies are needed to fully understand the complex nature of seizure genesis in MOGHE and TSC to inform optimal treatment.

## Conclusions

Collectively, clinical and modelling studies of MOGHE and FCD type 2 highlight both convergent and divergent mechanisms underlying epileptogenesis in each of these cortical malformations. Specifically, IUE-based models generating mice with cortex-specific mosaicism for either *Slc35a2* or mTOR signalling pathway genes display phenotypes with both shared and discrete characteristics. Shared features include altered neuronal migration and intrinsic excitability, whilst oligodendroglial hyperplasia is unique to MOGHE and dysmorphic neurons are a defining hallmark of mTORopathies. The functional consequences of glial involvement in MOGHE are relatively understudied compared to the role of *Slc35a2*-deficient excitatory neurons. Further investigation into how impaired N-glycosylation results in aberrant glial proliferation and activity may provide a promising avenue into better understanding of the pathogenesis of MOGHE and developing precision therapies such as ᴅ-galactose supplementation.

We have observed a convergent electrophysiological phenotype in excitatory neurons with *Slc35a2* and mTOR-pathway gene variants, namely that there is reduced action potential firing. Interestingly, this differs from most other epilepsy models, which show increased action potential firing. The significance of this remains unclear, with respect to how it relates to the clinical manifestations of these conditions and whether it plays a role in the emergence of infantile spasms, and also whether it suggests novel therapeutic approaches. Advancing therapeutic strategies will require a robust mechanistic understanding of how specific genetic defects drive pathogenesis, at cellular and network levels, and across development. Modelling studies in both rodents and iPSC-derived neuronal cultures have provided critical insights into MOGHE-related epilepsy to date, and will continue to be critical moving forward.

## Data Availability

No datasets were generated or analysed during the current study.
